# The *w*MelPop strain of *Wolbachia *interferes with dopamine levels in *Aedes aegypti*

**DOI:** 10.1186/1756-3305-4-28

**Published:** 2011-02-28

**Authors:** Luciano A Moreira, Yixin H Ye, Karly Turner, Darryl W Eyles, Elizabeth A McGraw, Scott L O'Neill

**Affiliations:** 1 School of Biological Sciences, The University of Queensland, Brisbane Qld 4072, Australia; 2 René Rachou Research Institute- FIOCRUZ, Belo Horizonte MG 30190, Brazil; 3 Queensland Brain Institute, The University of Queensland, Brisbane Qld 4072, Australia

## Abstract

*Wolbachia *is an intracellular bacterium that has been stably transinfected into the mosquito vector of dengue, *Aedes aegypti*. This inherited infection causes a range of metabolic and phenotypic alterations in the mosquito, which might be related to neuronal abnormalities. In order to determine if these alterations were caused by the manipulation of neuroamines by this bacterium, we studied the expression of genes involved in the dopamine biosynthetic pathway and also measured the amount of dopamine in infected and uninfected mosquitoes of different ages. *Wolbachia*-infected mosquitoes exhibit greater expression of some genes related to the melanization pathway, but not for those directly linked to dopamine production. Although dopamine levels were higher in *Wolbachia*-positive mosquitoes this was not consistent across all insect ages nor was it related to the previously described *Wolbachia *induced "bendy" and "shaky" phenotypes.

## Findings

*Aedes aegypti *is the main vector of dengue, one of the leading arboviral diseases of humans throughout the tropics of the world [1]. In an attempt to develop a new biocontrol approach to reduce dengue transmission, *A. aegypti *mosquitoes have been transinfected with the common inherited bacterial symbiont of insects, *Wolbachia *[2]. The wMelPop-CLA strain of *Wolbachia *when present in *A. aegypti *induces a range of effects including reductions in adult lifespan and blockage of vector competence for a range of human pathogens [2-5]. In addition some other effects have also been reported. Infected mosquitoes exhibit increases in both locomotor activity and metabolic rate [6]. As *Wolbachia*-infected *A. aegypti *females age, they obtain fewer and smaller blood meals and show increasing difficulty in completing the process of blood feeding [7,8]. Old female mosquitoes (>15 days) also display behavioral defects described as a "bendy" proboscis or jittering of the body termed "shaky", suggesting that their neuronal function is somehow impaired.

The biogenic amines dopamine, serotonin and octopamine act as signaling molecules in many diverse physiological contexts, including behavior, fertility, and reproduction, and in the development of neuronal and non-neuronal tissues [9]. In insects, dopamine is involved in several other biological processes, that span from cuticle formation [10], to egg chorion hardening [11], to gonadotropic regulation [12] and to the immune response [13,14]. Dopamine is associated with locomotor defects in Drosophila [15] and mammals [16] and, in humans its deficiency is the cause of motor defects such as Parkinson disease [17].

**Figure 1:**
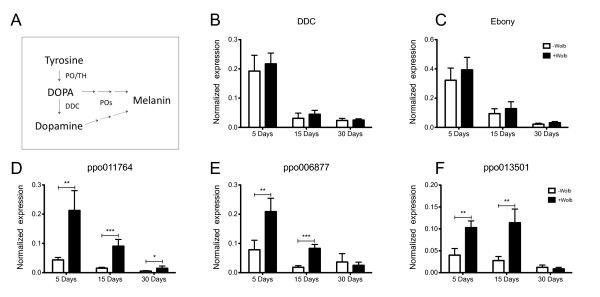
**Expression analysis of genes involved in the dopamine pathway**. (A) Simplified dopamine pathway involving the enzymes phenoloxidase (PO), Tyrosine hydroxylase (TH) and DOPA-decarboxylase. (B-F) Quantitative PCR of Ebony, DDC and ppo genes in *Wolbachia*-infected (Wolb+) and uninfected (Wolb-) *A. aegypti *mosquito heads of three different ages (5, 15 and 30 days-old) (n = 5 for each time point). *P < 0.05, **P < 0.01 and ***P < 0.001.

Many genes participate in the dopamine biosynthesis pathway, which is highly conserved among vertebrates and invertebrates. Initially, the enzyme tyrosine hydroxylase (TH) [or phenoloxidase - (PO)] catalyzes the conversion of tyrosine to L-DOPA [14,18], which is then decarboxylated by the enzyme dopa-decarboxylase (DDC) to dopamine (DA)[9] (Figure 1A). In *Drosophila*, the Ebony protein, which is required for normal behavioral rhythmicity [19] has also been shown to control (through a β-alanyl-dopamine synthase activity) the dopamine pathway [20].

To examine a possible relationship between dopamine production and the previously described behavioral defects in mosquitoes associated with *Wolbachia *infection, we characterized transcription of a number of genes involved in the dopamine biosynthetic pathway [21] (Figure 1A) in mosquito heads at 3 different adult ages. *A. aegypti *mosquitoes, *w*MelPop-CLA infected and tetracycline cured strains [2], were reared in a controlled environment insectary at 26°C and approx. 80% humidity. All experiments were carried out on sucrose fed individuals. Thirty day-old *Wolbachia*-infected females were sorted into two different phenotypic categories - normal and abnormal (containing "shaky" or "bendy") by placing insects into a cage and observing their probing behavior on a human hand [7].

For gene expression studies pools containing 20 female mosquitoes were dissected in ice-cold PBS, pooled, snap frozen in liquid nitrogen and extracted for total RNA using Trizol (Invitrogen Corp., Carlsbad, CA). Total RNA was treated with 2 μL of DNase I (Roche) for 30 minutes at 37°C in a 20 μL reaction to eliminate genomic DNA. Approximately 0.5 μg of total RNA was reverse transcribed using random primers and SuperScript III reverse transcriptase (Invitrogen) according to manufacturer's protocols.

Quantitative real-time PCR (qRT-PCR) was used to measure the relative expression of a set of target genes that have been shown to be involved in dopamine synthesis and elimination (Figure 1A; Table 1). *A. aegypti ribosomal protein S17 *(*Ae-RpS17*, Table 1) was used as a reference gene [22] and the single copy *Wolbachia *ankyrin repeat gene (WD0550) (ANK550, Table 1) was used to calculate the density of bacteria in individual mosquitoes when normalized to the S17 gene. RT-qPCR was performed on a Rotor-gene 6000 (Corbett Life Science, Sydney, NSW) using Platinum^®^SYBR^®^Green (Invitrogen Inc, Carlsbad, CA) according to the manufacturer's instructions. For each sample a mastermix of 2 μL RNase-free water, 5 μL of SYBR Supermix and 0.5 μL of each primer (5 μM) were added to 2 μL of cDNA or gDNA. Three technical replicates were run for each sample. The cycling protocol was as follows; 2 minutes at 50°C for the UDG incubation, 2 minutes at 95°C for *Taq *activation, 40 cycles of denaturation at 95°C for 5 s, annealing at 60°C for 5 s and extension and fluorescence acquisition at 72°C for 15 s and then a melt curve analysis from 68-95°C in 1°C increments. The mean Cycle Threshold (CT) and mean amplification efficiency (E) per biological replicate was calculated from the three technical replicates using Rotor-Gene 6000 Series Software ver.1.7.75 (Info-ZIP Pty Ltd.). The raw output CT data was normalized by taking into consideration the differences in amplification efficiency of target and the reference genes using Q-gene software [23]. The data were then analysed using general linear models with age (5,15 or 30 days) and line (+Wolb or -Wolb) as factors using Statistica 8.0 (StatSoft, Inc.).

**Table 1:** Primers used for quantitative PCR analysis of genes related to dopamine metabolism

Accession	Primer	Sequence 5'-3'	Amplicon (bp)
AAEL004175-RA	Ae-RpS17F	CACTCCCAGGTCCGTGGTAT	81
	Ae-RpS17R	GGACACTTCCGGCACGTAGT	
AAEL014238-RB	Ddc F	GGTGGACTACATCGCCAACT	166
	Ddc R	GACACCAGGCATGATGACAC	
			
AAEL005793-RA	Ebony F	GAATCGGGACGGAGATTACA	198
	Ebony R	ACGGCCACATTTTCGTAGTC	
			
AAEL011764-RA	Ppo011764 F	CTGAACAACGGATTCCCATT	165
	Ppo011764 R	TCATCAACGTCTGCACATCA	
			
AAEL006877-RA	Ppo006877 F	CTATACGGCTTCGGATCGAG	184
	Ppo006877 R	GTACCGAGCCATCGTTTGTT	
			
AAEL013501-RA	Ppo013501 F	CTTCGGATCGAGAGCTTGAG	176
	Ppo013501 R	GTACCGAGCCATCGTTTGTT	
NC_002978: c537094-538083	ANK550 F	CAGGAGTTGCTGTGGGTATATTAG	74
	ANK550 R	TGCAGGTAATGCAGTAGCGTAAA	

Expression of all genes reduces with age, regardless of infection status (Figure 1; see Table S1; Additional file 1). However expression is significantly higher in *Wolbachia *infected mosquitoes for the three-prophenoloxidase (ppo) genes, but not for DDC or Ebony (Figure 1B-F). The increased expression of genes related to the melanization pathway in *Wolbachia *infected insects is consistent with the finding that several immunity genes (including AAEL011764) are also up-regulated by the presence of this bacterium in *A. aegypti *[3,4] and more recently that *Wolbachia*-infected mosquitoes exhibit higher levels of melanization in their hemolymph [24].

Dopamine levels were also determined in mosquitoes of three ages (5, 15, and 30 days). Our hypothesis was that *Wolbachia*-infected mosquitoes, would exhibit lower levels of dopamine, resembling the situation in Parkinson's Disease in humans [17]. Mosquitoes were anesthetized with CO_2 _and then decapitated. Preliminary analysis indicated pooling 5 heads was sufficient to achieve a robust dopamine signal and hence all reported sample sizes represent pools of 5 heads. Heads were snap frozen on dry ice and kept at -80°C for up to two weeks. One hundred microliters of 0.1 M perchloric acid containing 5 ng deoxyepinephrine (as an internal standard) was added to each tube. Heads were sonicated using a probe setting of 60% amp/20 Khz for 5 sec, followed by 10 sec rest and another 5 sec sonication (on ice). Tubes were centrifuged for 5 min at 13,000 rpm. Supernatant was filtered through a 4 mm/0.22 syringe filter (PM Separations, Australia) and injected into a HPLC system that consisted of a degasser, autosampler and an isocratic pump (Model 1100, Agilent Technologies, Inc. CA), a Sunfire C18 column, 4.6 mm × 150 mm, 5 um; (Waters Corporation, MA) and a Coulochem III (ESA Laboratories, Inc. MA) electrochemical detector. The mobile phase was a 12% acetonitrile/75 mM potassium dihydrogen phosphate buffer containing 1 mM EDTA and 1.4 mM octane sulfonic acid adjusted to pH 4.13 with phosphoric acid. Flow rate was 1.2 ml/min. The conditioning cell (Model 5020, ESA Laboratories, Inc. MA) operated at +350 mV with the first and second electrode of the analytical cell (Model 5014B, ESA Laboratories, Inc. MA) potentials set to -150 mV and +250 mV respectively. Data were quantified by calculating peak-height ratios for dopamine relative to deoxyepinephrine, and calibrated using a standard curve. Data were then processed with Chemstation software (Rev B.01.03, Agilent Technologies, Inc. CA). Samples were corrected for dilution and expressed as pg/mosquito head. Dopamine levels were analysed using standard t-tests comparing the effect of line (+Wolb or -Wolb) at each of three adult ages (5,15 or 30 days) using Statistica 8.0 (StatSoft, Inc.). In each case comparisons were only made between mosquitoes collected on the same day and analysed in the same batch.

**Figure 2:**
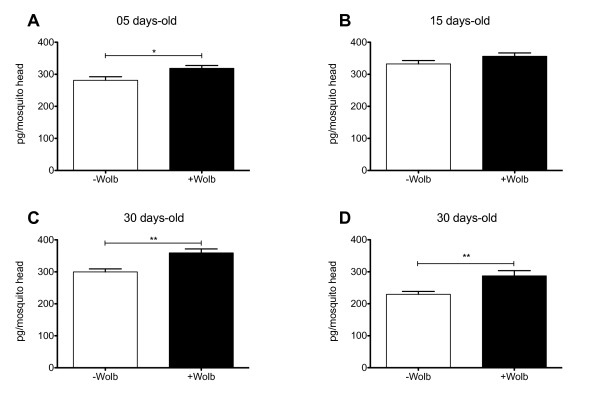
**Dopamine levels in mosquito heads**. (A-D) Dopamine levels (pg/mosquito head) in *Wolbachia *infected (+ Wolb) and uninfected *A. aegypti *mosquitoes (- Wolb) of three different ages (5, 15 and 30 days-old). Sample sizes were as follows with each n = pool of 5 heads; 5 days n = 40, 15 days n = 26, 30 days n = 22-42. On D a replicate experiment is shown. *P < 0.05, **P < 0.01.

**Figure 3:**
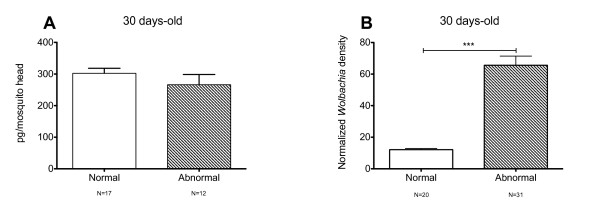
**Dopamine levels in phenotyped mosquitoes**. (A) Dopamine levels in 30 days-old *Wolbachia*-positive mosquitoes exhibiting normal or abnormal behavior ("shaky" or "bendy", see text) (n = number of pools). (B) Normalized *Wolbachia *density (ankirin gene: RPS17, see text) in individual mosquitoes exhibiting normal or abnormal behavior (n = number of individual females). ***P < 0.0001.

The levels of dopamine reported here (2.2 pMol/head) are in keeping with previously reported measures for female *A. aegypti *heads [25]. Dopamine levels were significantly higher in five and 30 day-old mosquitoes when *Wolbachia *is present (Figure 2A,C). As we have previously seen abnormal phenotypes in older mosquitoes (> 15 days-old) we further analyzed dopamine contents in normal and *Wolbachia*-infected mosquitoes that exhibited these phenotypes (shaky or bendy). Results show that there was no difference between normal and abnormal mosquitoes (Figure 3A), although *Wolbachia *densities were higher in abnormal mosquitoes (Figure 3B). We can conclude then that dopamine levels alone cannot explain the phenotypes. Artificial manipulation of dopamine levels by chemical means in *A. aegypti *could be employed in future studies in an attempt to determine what if any effect the *Wolbachia*-induced elevation in dopamine is having on the biology of the insect.

Taken together, our findings indicate that *Wolbachia*-infected mosquitoes have higher dopamine levels in their heads, although the effect was not present at all ages examined. These higher dopamine levels are consistent with previous observation that *Wolbachia*-infected mosquitoes exhibit higher activity [6]. There may be other behaviours governed by dopamine that are also affected by *Wolbachia *infection however there is no obvious correlation between higher dopamine levels found here and the abnormal behavioral phenotypes "shaky" and "bendy" we examined. The positive correlation between *Wolbachia *densities and prevalence of behavioral phenotypes suggests that bacterial densities are predictive of virulence.

## Competing interests

The authors declare that they have no competing interests.

## Authors' contributions

LAM conceived the studied, helped on the dopamine assays and drafted the manuscript. YE carried out the gene expression studies. KT performed the dopamine assays. DE participated in the design of the study and helped to draft the manuscript. EAM helped on statistical analysis and to draft the manuscript. SLO conceived of the study, participated in its design and coordination and helped to draft the manuscript. All authors read and approved the final manuscript.

## Supplementary Material

Additional file 1 **Statistical analysis of gene expression**. Table S1 shows the analysis of quantitative expression of mosquito genes related to dopamine pathway Click here for file
