# Supplementary material for: The wMelPop strain of Wolbachia interferes with dopamine levels in Aedes aegypti
Source: Parasit Vectors. 2011 Feb 28;4:28. doi: 10.1186/1756-3305-4-28 (PMC3058110; doi:10.1186/1756-3305-4-28)
Supplement: Additional file 1 — Statistical analysis of gene expression. Table S1 shows the analysis of quantitative expression of mosquito genes related to dopamine pathway [file 1756-3305-4-28-S1.DOC]

**Table S1.** Statistical analysis (t-tests) of quantitative expression of mosquito genes, related to dopamine pathway.

| Gene | Age (days) | Pools (n) | *df* | F value | *P* value |
| --- | --- | --- | --- | --- | --- |
| Ddc | 5 | 5 | 8 | 2.122243 | 0.425920 |
|  | 15 | 5 | 8 | 1.746789 | 0.202109 |
|  | 30 | 5 | 8 | 2.884879 | 0.700974 |
| Ebony | 5 | 5 | 8 | 1.060656 | 0.217703 |
|  | 15 | 5 | 8 | 1.971665 | 0.220743 |
|  | 30 | 5 | 8 | 1.634231 | 0.079692 |
| Ppo011764 | 5 | 5 | 8 | 61.82240 | 0.000543 |
|  | 15 | 5 | 8 | 60.40788 | 0.000086 |
|  | 30 | 5 | 8 | 27.85682 | 0.023923 |
| Ppo006877 | 5 | 5 | 8 | 1.967591 | 0.000839 |
|  | 15 | 5 | 8 | 5.903425 | 0.000009 |
|  | 30 | 2-5 | 5 | 6.731335 | 0.425174 |
| Ppo013501 | 5 | 5 | 8 | 1.020245 | 0.000173 |
|  | 15 | 5 | 8 | 11.26505 | 0.000348 |
|  | 30 | 5 | 8 | 97.10637 | 0.003851 |
